# Biomedical retrieval-augmented generation for relation classification

**DOI:** 10.3389/frma.2026.1919972

**Published:** 2026-09-29

**Authors:** Jannat, Charlie Dil, Tom Arodz, Bridget T. McInnes

**Affiliations:** Department of Computer Science, Virginia Commonwealth University, Richmond, VA, United States

**Keywords:** biomedical relation classification, few-shot learning, GutBrainIE, large language models, out-of-schema prediction, Qwen, relation classification, retrieval-augmented generation

## Abstract

The rapid expansion of biomedical literature requires automated methods for accurate and efficient information extraction. This study addresses relation classification: given a pair of annotated biomedical entities in a research article title and abstract, assigning the relation that holds between them from a pre-defined set. We evaluate a retrieval-augmented generation (RAG) framework in which semantically similar annotated examples are retrieved from a training pool and supplied to a large language model as in-context demonstrations. Four open-source LLMs are compared against a supervised DeBERTa+CNN baseline on GutBrainIE, and four further corpora (ChemProt, BioRED, its BioCreative VIII release BC8, and DDI-2013) are used to test whether the findings generalize. We report macro- and micro-averaged precision, recall and F1-score, and the rate of predictions that fall outside the defined relation set. On GutBrainIE, Qwen3:14B with 25 retrieved examples reaches 0.877 macro and 0.866 micro F1, against 0.440 and 0.562 for the supervised baseline, while the smaller Llama-3.1-8B and Gemma3:12B models do not exceed the baseline on macro F1. Macro F1 rises from 0.105 without demonstrations to 0.877 with 25, with the steepest gains over the first ten retrieved examples and subsequent variation smaller than the run-to-run standard deviation. Predictions falling outside the defined relation set drop from 39 at zero shots to at most one between 20 and 35, so retrieval improves output validity as well as accuracy. Across the four further corpora these gains do not generalize uniformly: the best configuration exceeds the supervised baseline on ChemProt, improves macro but not micro F1 on BioRED and BC8, and falls below it on both measures on DDI-2013. Since DDI-2013 is the most balanced corpus in the set and, like ChemProt, is annotated within single sentences, that reversal is not explained by class imbalance or annotation scope, but is consistent with what the relation labels encode: whether they name relationships recoverable from biomedical knowledge, or boundaries set by an annotation guideline.

## 1 Introduction

The rapid growth of electronic texts has made it increasingly challenging to identify and synthesize relevant knowledge efficiently. This challenge is particularly pronounced in the biomedical domain, where the volume of scientific literature continues to grow at an accelerating rate. As a result, automated approaches for extracting structured information from unstructured text are essential.

Information extraction (IE) is a core task in natural language processing which focuses on identifying structured information from text within a specific domain (Hobbs and Riloff, 2010). The demand for IE from large-scale scientific corpora has grown substantially in recent years (Brokman et al., 2025). Relation extraction (RE) and classification, a key subtask of IE, aims to identify semantic relationships between entities and represent them in structured forms such as knowledge bases (Wang et al., 2022). In the biomedical domain, RE supports a range of applications, including the identification of drug-drug interactions (DDIs), which describe how the effect of one drug may be altered by another (Baxter, 2010). Accurate extraction and classification of such relationships from scientific literature is critical for supporting research, clinical decision-making, and knowledge discovery.

Traditional RE approaches rely on supervised models that are fine-tuned on domain-specific datasets. However, these models are often limited by the scope of their training data and can struggle to generalize to newly published findings. Additionally, updating such models to incorporate emerging knowledge can be computationally expensive and time-consuming. More recently, large language models (LLMs) have demonstrated strong performance across a range of natural language tasks through prompt-based learning (Mann et al., 2020). Techniques such as few-shot prompting and chain-of-thought reasoning enable LLMs to adapt to new tasks without explicit retraining. Despite these advances, LLMs often underperform on biomedical tasks, where domain-specific knowledge is critical and large-scale fine-tuning is constrained by limited labeled data (Nagar et al., 2025).

Retrieval-augmented generation (RAG) (Lewis et al., 2020) has emerged as a promising approach to address these limitations by incorporating external knowledge into the generation process. In RAG frameworks, relevant information is dynamically retrieved from external sources, such as vector databases, and integrated into prompts provided to LLMs. This approach improves factual grounding, reduces hallucinations, and enhances robustness when dealing with complex or domain-specific queries.

In this work, we investigate the use of retrieval-augmented generation for biomedical relation classification, that is, assigning a relation from a pre-defined set to a pair of entities supplied with the text. Our approach augments large language models with retrieved, domain-specific examples drawn from a reference corpus, allowing the model to leverage both contextual reasoning and external knowledge during inference. Prior studies have shown that RAG can mitigate hallucinations and improve reasoning in information extraction tasks (Chen et al., 2024; Olasunkanmi et al., 2025; Lewis et al., 2020).

Building on these developments, this study aims to evaluate the effectiveness of RAG-based pipelines for biomedical relation classification and to better understand the role of in-context learning. Specifically, we address the following research questions:

- How does the performance of RAG-based biomedical relation classification vary across LLMs relative to a DeBERTa + CNN and LLM alone without providing relevant examples as baseline in terms of precision, recall, and F1-score?
- What is the impact of increasing in-context examples on performance, and where do diminishing returns occur for different LLMs?
- To what extent does retrieval augmentation reduce out-of-schema or incorrect relation labels in LLM-based classification?
- Does the benefit of retrieval augmentation generalize across corpora with different relation schemas, and what distinguishes corpora where it helps from those where it does not?

Qur contributions are fourfold. First, we compare four open-source LLMs under retrieval-augmented prompting against a supervised DeBERTa+CNN baseline on GutBrainIE, and find that Qwen3:14B exceeds the baseline substantially on both macro and micro F1 while the smaller Llama-3.1-8B and Gemma3:12B models do not exceed it on macro F1. Second, through a shot ablation with replicated measurements we characterize the data-efficiency of in-context retrieval: performance rises steeply within the first 10 retrieved examples, continues to improve to 25, and thereafter varies by less than the run-to-run standard deviation. Third, we quantify how retrieval augmentation reduces predictions that fall outside the defined relation set, sharply at low shot counts. Fourth, we test whether these findings generalize by repeating the comparison on four additional datasets and find that the benefit of retrieval is not uniform. It holds on ChemProt, where the retrieval-augmented configuration again exceeds the baseline on both measures. On BioRED and its BioCreative VIII release it improves macro but not micro F1, and on DDI-2013 the comparison reverses entirely, which we trace to what the relation labels encode. Unlike prior RAG-for-relation-classification work, our emphasis is this controlled analysis of when in-context grounding helps, rather than proposing a new retrieval architecture.

## 2 Related work

The evolution of Retrieval-Augmented Generation (RAG) has significantly reshaped knowledge-intensive Natural Language Processing (NLP). Traditional language models rely primarily on parametric knowledge encoded in their weights, limiting their ability to update information and increasing susceptibility to outdated or hallucinated responses. In contrast, RAG integrates external, updatable knowledge sources, enabling models to ground their outputs in retrieved evidence and support more reliable reasoning.

Lewis et al. (2020) formalized the RAG framework by combining parametric memory (stored within model weights) with non-parametric memory (retrieved from external corpora). In this architecture, a retriever selects relevant documents, which are then passed to a generator, implemented using BART, to produce the final output. This approach builds on Dense Passage Retrieval (DPR) (Karpukhin et al., 2020), which employs dual encoders to independently represent queries and passages, enabling efficient dense retrieval. Evaluations on benchmarks such as Natural Questions, TriviaQA, WebQuestions, CuratedTREC, and MS MARCO demonstrate consistent improvements across standard metrics, including exact match and F1 score.

Beyond performance gains, RAG reduces hallucination by grounding generation in retrieved context through non-parametric memory. DPR (Karpukhin et al., 2020) has become a foundational component of modern retrieval-based pipelines, offering a strong alternative to classical sparse retrieval methods (e.g., TF-IDF and BM25) by improving top-*k* recall and semantic matching.

The motivation for RAG arises from limitations in purely parametric models such as GPT-3 (Brown et al., 2020) and LLaMA (Touvron et al., 2023). While these models exhibit strong generalization capabilities, they require costly retraining to update knowledge and are prone to generating plausible but incorrect information, a phenomenon known as hallucination (Li et al., 2024).

More broadly, recent work on RAG has focused on four main directions: (1) improved generator architectures, (2) more effective retrieval strategies, (3) enhanced augmentation techniques involving prompts, queries, and data, and (4) more rigorous evaluation frameworks for assessing reliability, adaptability, and domain-specific performance.

As retrieval serves as the backbone of RAG, it directly impacts LLM performance through the relevance and quality of the context provided to the generator. Recent approaches have moved beyond standard sparse, keyword-based pipelines toward more advanced strategies, including dense, hybrid, multi-hop, and reranking-based retrieval. The effectiveness of these methods is typically evaluated using metrics such as recall, precision, and robustness, as summarized in Table 1.

**Table 1:** Results and impact of retrieval strategies in retrieval-augmented generation (RAG) systems.

Paper	Approach	Results	Dataset
Dense passage retrieval (DPR) (Karpukhin et al., 2020)	Dense dual-encoder retrieval replacing sparse BM25	Top-20 recall: 85.4% (vs. BM25: 64.2%), EM ≥ 42%, F1 +9–11%	Natural questions (NQ), TriviaQA, WebQuestions
HYBGRAG (Lee et al., 2024)	Hybrid (dense FAISS, sparse BM25, ensemble fusion, reranking)	Precision@6: 88% (+10% vs. single retriever), higher recall, increased computation	Multi-hop QA
Re2G (Glass et al., 2022)	Retrieve, rerank (neural), generate	Up to 34% gain in multi-hop QAs, better output through relevant reranking	ARC Challenge, KILT T-REx
RQ-RAG (Chan et al., 2024)	LLM-based query refinement before retrieval	F1 improvement: +6-12% in multi-hop/ambiguous QA; higher complex/reasoning relevance	PubHealth, ARC Challenge
RELATE (Olasunkanmi et al., 2025)	Ontology-constrained biomedical relation extraction combining vector search and LLM reranking	52% exact match and 94% accuracy@10 on ChemProt; effectively filters implausible relations	ChemProt, HEAL project abstracts
LLMs are not zero-shot reasoners (Nagar et al., 2025)	Systematic benchmark of LLMs for biomedical IE with CoT, self-consistency, and RAG	Shows that standard prompting often outperforms CoT/RAG; highlights limitations of current zero-shot reasoning for biomedical IE	Multiple biomedical classification and NER datasets
SyRACT (Dong et al., 2025)	Synergistic RAG + Chain-of-Thought for zero-shot BioDocRE	Improves F1 by 11.0, 9.1, and 41.0% on CDR, GDA, and ADE compared to DocRE with standard prompts	CDR, GDA, ADE
Dynamic parametric RAG (DyPRAG) (Tan et al., 2025)	Dynamic injection of docs into LLM parameters at test time	Latency reduction: 12%, accuracy improvement: 15%, better generalization	Open-domain QA
GraphRAG (Han et al., 2024), GraphRAG-Bench (Xiao et al., 2025)	Graph-based retrieval enabling multi-hop, structured reasoning	Accuracy: 80%–90% (vs. 50%–67% for classic RAG); best for complex queries	Industrial/tech datasets

Dense Passage Retrieval (DPR) (Karpukhin et al., 2020) replaces traditional sparse retrieval methods such as BM25 (Robertson et al., 2009) with dual-encoder dense embeddings for both queries and passages. In open-domain question answering, DPR achieves a top-20 passage recall of 85.4% on Natural Questions (NQ), compared to 64.2% for BM25, yielding an absolute improvement of 21.2%. When integrated into a RAG pipeline, DPR-based systems achieve Exact Match (EM) scores exceeding 42% and F1 improvements of 9-11% across major QA benchmarks, demonstrating the effectiveness of dense retrieval for high-quality context selection.

Hybrid retrieval strategies such as HYBGRAG (Lee et al., 2024) combine dense retrieval (e.g., FAISS-based embeddings), sparse retrieval (BM25), and multi-query expansion. These systems often incorporate ensemble fusion techniques and reranking methods such as Maximum Marginal Relevance (MMR), improving retrieval diversity and relevance at the cost of increased computational overhead. Similarly, Re2G (Glass et al., 2022) improves retrieval quality by combining dense and sparse retrieval outputs followed by neural reranking before generation, achieving up to 34% improvement on multi-hop reasoning tasks. These results highlight the consistent benefit of reranking mechanisms in improving retrieval relevance and downstream generation quality.

Query reformulation approaches such as RQ-RAG (Chan et al., 2024) train large language models to rewrite, decompose, or disambiguate user queries prior to retrieval. This addresses a key limitation of standard RAG pipelines, which rely directly on raw user input. By improving query clarity, RQ-RAG increases retrieval relevance for ambiguous or complex multi-hop questions, leading to improved overall QA performance.

Dynamic Parametric RAG (DyPRAG) (Tan et al., 2025) introduces a framework in which external knowledge is incorporated into model inference through dynamic parameter adaptation during test time. This reduces reliance on static model parameters and can improve both latency and storage efficiency compared to conventional retrieval pipelines. DyPRAG demonstrates improvements in knowledge integration and hallucination reduction, with strong generalization across QA benchmarks.

Graph-based retrieval methods such as GraphRAG (Han et al., 2024) and GraphRAG-Bench (Xiao et al., 2025) extend retrieval beyond unstructured text by constructing graph representations for multi-hop and compositional reasoning. Empirical results on industrial and technical benchmarks show substantial performance gains over standard RAG approaches, particularly for complex queries requiring multi-step reasoning. In these settings, GraphRAG achieves accuracies of 80-90%, compared to 50-67% for traditional RAG pipelines, highlighting its effectiveness in structured reasoning tasks.

Retrieval-augmented prompting is one of several methods to adapt a general-purpose language model to a specific task or domain, and it is useful to state what it is being compared against. Supervised fine-tuning updates model weights on labeled data and remains the strongest option when a sufficient number of annotated examples are available (Devlin et al., 2019); its costs are the labeled data itself which can be both time consuming and expensive, the compute to train, and a separate set of weights per task and per schema. In-context learning leaves weights unchanged and supplies demonstrations in the prompt (Brown et al., 2020); it requires no training but is bounded by the context window and by what a handful of examples can convey. Model steering intervenes on internal activations at inference time to bias generation toward a desired behavior, occupying a middle ground in which weights are fixed but the forward pass is modified (Subramani et al., 2022).

Retrieval-augmented prompting is a form of in-context learning in which the demonstrations are selected per query rather than fixed. The present study is therefore a controlled in-context learning experiment: the zero-shot condition is in-context learning with no demonstrations, the shot sweep varies the number of demonstrations directly, and the supervised DeBERTa+CNN baseline is the fine-tuning comparator. What the cross-corpus results show is where the boundary between these two regimes falls.

Beyond general domain question answering, RAG has increasingly been adapted to biomedical information extraction and classification tasks, where domain ontologies and safety constraints are critical. RELATE (Olasunkanmi et al., 2025) combines SapBERT–enhanced vector search with LLM–based reranking under ontology constraints, achieving 52% exact match and 94% accuracy@10 on ChemProt and effectively filtering implausible relations in 2,400 HEAL project abstracts. Nagar et al. (2025) systematically benchmark LLMs for biomedical classification and NER with chain–of–thought prompting, self–consistency, and RAG, showing that standard prompting often outperforms these more complex setups and concluding that current LLMs are not reliable zero–shot reasoners for structured biomedical IE. Complementing these negative results, SyRACT (Dong et al., 2025) frames document–level relation extraction as a QA problem and synergistically combines RAG with task–specific chain–of–thought, yielding F1 gains of 11.04, 9.10, and 41.00% on CDR, GDA, and ADE respectively over DocRE baselines that use standard prompts. In contrast to these methods, which contribute new retrieval or prompting *architectures* evaluated on document–level or chemical-protein corpora, our focus is a controlled empirical analysis of how the *number* of retrieved in–context examples affects relation–classification accuracy and out-of-schema predictions on a public sentence–level benchmark, across models of differing scale.

Overall, these retrieval strategies (as summarized in Table 1) demonstrate that RAG systems have evolved from simple sparse retrieval pipelines to more sophisticated architectures incorporating dense retrieval, hybrid fusion, reranking, query reformulation, and graph-based reasoning. These advances enable LLMs to retrieve, filter, and integrate external knowledge more effectively, improving robustness, coverage, and performance on complex question answering tasks.

The BioRED corpus and its BioCreative VIII release served as the basis for a community shared task, with the participating systems establishing the strongest published benchmarks for this dataset (Islamaj et al., 2024). The three highest-ranked teams proposed ensembles of domain-pre-trained encoders (rather than prompting). The leading system combined PubMedBERT with coreference resolution, adversarial training and multitask learning over relation extraction and novelty detection; a third-ranked system ensembled PubMedBERT models with a max-rule aggregation over extensively pre-processed input. Closest to the present work is the second-ranked system of Phan et al. (2024), which uses general-purpose LLMs alongside supervised models. Specifically, GPT-4 and Gemini generate augmented training data and provide a complementary classification signal, which is then ensembled with fine-tuned PubMedBERT classifiers. To contrast with our setting, there, large language models serve a supervised pipeline by creating synthetic training data; here, they perform classification directly from retrieved examples with no parameter updates. The two are complementary, and the augmentation strategy of Phan et al. provides an intuitive solution for addressing specific datasets or relations where retrieval-augmented prompting underperforms. Shared-task scores are not directly comparable to ours. The BioCreative VIII task is end-to-end, requiring entity recognition, relation typing and novelty prediction, whereas we classify relations over gold entity pairs. The reported F1 of roughly 0.44 for the leading system therefore measures a substantially harder task than the one evaluated here.

## 3 Materials and methods

This section describes the design and implementation of our retrieval-augmented generation (RAG) pipeline for biomedical relation classification. The system integrates dense retrieval of relevant training examples with large language model inference, enabling accurate identification of relationships between entities in biomedical text. The pipeline consists of four main components: (i) embedding generation, (ii) vector database indexing, (iii) query processing and retrieval, and (iv) prompt construction for LLM-based relation prediction. Each component is described in detail in the subsections below.

### 3.1 Embedding

All training samples are converted into document objects with associated metadata and processed through LlamaIndex's ingestion pipeline. To enhance retrieval quality and contextual understanding, each document chunk, consisting of sentences from article titles and abstracts together with their entity annotations, is encoded into a single dense 384-dimensional vector using the HuggingFaceEmbedding interface with the all-MiniLM-L6-v2 model. We use a general-purpose sentence encoder rather than a biomedical-specific one for its strong retrieval performance, low latency, and broad adoption; the contrast with domain-specific embedding is left to future work. The full input-to-embedding pipeline (chunking and encoding) is illustrated in Figure 1.

**Figure 1:**
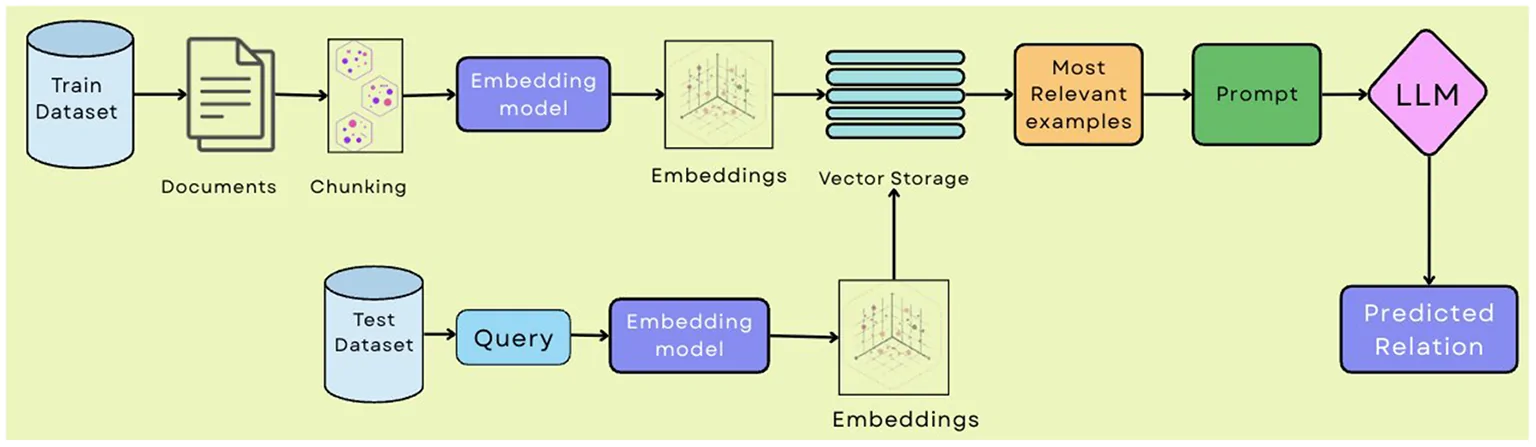
Methodology: RAG for Relation Classification on biomedical dataset.

Embeddings are generated for both training and evaluation datasets. During inference, each item from the development dataset serves as a query (Figure 1). These embeddings form the foundation for retrieval and inference, ensuring that semantically similar sentences are effectively matched and leveraged by the generative model.

### 3.2 Vector indexing and retrieval infrastructure

After generating embeddings for all training documents, each embedding is mapped into a continuous vector space to enable semantic similarity search. These embeddings are stored and indexed in a vector database, which allows efficient retrieval of relevant examples at inference time. In this work, we use Qdrant as the vector store, integrated via the QdrantVectorStore module in LlamaIndex (Ockerman et al., 2025). Qdrant is optimized for high-performance semantic search and approximate nearest neighbor retrieval, enabling rapid identification of the most similar training instances for a given query.

This vector-based retrieval approach allows the system to match new queries with semantically relevant examples from the training dataset by cosine similarity in embedding space rather than by lexical overlap. By capturing the underlying semantic relationships between biomedical sentences, the vector index ensures that the RAG pipeline can provide contextually appropriate examples to the LLM.

### 3.3 Query representation and embedding

During inference, each test sample is treated as a query. The input text, which includes sentences and entity annotations, is encoded into a dense vector embedding using the same embedding model employed for the training data (all-MiniLM-L6-v2). These query embeddings are not stored in the vector database; instead, they are used dynamically to identify the most semantically similar training instances.

By leveraging LlamaIndex's abstraction layer, queries are seamlessly converted into embeddings and passed to Qdrant for similarity search. This process ensures that each query is compared in the same vector space as the indexed training embeddings, enabling retrieval of contextually relevant examples. Using this approach, the RAG pipeline can supply the LLM with domain-specific, semantically aligned examples at inference time.

### 3.4 Retrieval of relevant examples

In the retrieval step, both query embeddings and training embeddings are represented in the same dense vector space using the all-MiniLM-L6-v2 model. This ensures that semantic similarity can be directly computed between the query and each stored training example, enabling the system to identify the most contextually relevant biomedical sentences.

For each query, the top-*k* most similar training nodes are retrieved by cosine similarity. Since all embeddings are *L*_2_-normalized, cosine similarity reduces to the inner product (Equation 1):


$$\begin{array}{l} \text{sim}\left(\text{q},\text{d}\right)=\frac{\text{q}\cdot \text{d}}{∥\text{q}∥∥\text{d}∥}=\text{q}\cdot \text{d},\;∥\text{q}∥=∥\text{d}∥=1, \end{array}$$ (1)

where **q** is the query embedding and **d** a stored training-example embedding. Retrieval is performed with Qdrant's Distance.Cosine metric. These retrieved examples serve as few-shot demonstrations for the generative model, providing domain-specific context that guides relation classification. Each retrieved node includes the passage text, entity annotations, labels, and relation information, which are incorporated into a structured prompt for the LLM.

This structured prompt ensures that the LLM has access to relevant, high-quality examples during inference. The overall retrieval and prompt construction process is illustrated in Figure 1, showing how query embeddings are mapped to the vector space, matched with training examples, and formatted for generation.

### 3.5 Prompt construction and LLM generation

The final step of the RAG pipeline is generation, where retrieved examples are incorporated into structured prompts and fed to large language models (LLMs) to predict relations between biomedical entities. Each model receives a context-enriched prompt containing the top-k retrieved examples along with the query sentence. The prompt instructs the model to extract and, when applicable, explain the relationship between the given entities. Because the head and tail entities are supplied in the prompt, the task is framed as relation *classification* over the pre-defined GutBrainIE relation set rather than end-to-end extraction; this isolates each model's relation-typing ability given fixed entity pairs. The complete prompt template and a worked example (with a model rationale) are provided in the Supplementary material S1, S2.

To evaluate retrieval-augmented prompting across open-source LLMs, we compare four configurations spanning different model scales and a controlled reasoning contrast. The Qwen3:14B model can be run with or without explicit chain-of-thought (“thinking”) generation, giving a matched reasoning vs. non-reasoning pair on identical weights; Llama-3.1-8B-Instruct and Gemma3:12B provide additional comparison points at smaller and intermediate scales. All are instruction-tuned, served locally via Ollama, and prompted identically.

- **Llama-3.1-8B-Instruct** (Touvron et al., 2023), a compact (8B) instruction-tuned model from the Llama family, included to gauge how smaller generalist models handle domain-specific relation classification with retrieval-augmented prompts.
- **Gemma3:12B** (Team et al., 2024), an open 12B instruction-tuned model from Google, included to broaden the comparison at an intermediate scale.
- **Qwen3:14B (non-thinking)** (Bai et al., 2023), the Qwen3 14B model run *without* explicit reasoning generation, producing only the predicted relation label.
- **Qwen3:14B (thinking)**, the same Qwen3 14B model run *with* chain-of-thought (“thinking”) enabled, producing a short rationale alongside the relation label. Because the weights are identical, the thinking/non-thinking pair isolates the effect of explicit reasoning.

All models were integrated into the RAG pipeline using LlamaIndex and Ollama infrastructure. Each model received the same structured, context-enriched prompts and produced predictions in a standardized JSON format. By systematically varying the number of retrieved examples included in the prompt, we evaluated each model's accuracy, prediction quality, and robustness against ground-truth biomedical relationships from the dataset.

## 4 Experimental setup

### 4.1 Dataset

The dataset for this work is derived from the BioASQ Conference and Labs of the Evaluation Forum (CLEF) 2025 GutBrainIE challenge (Martinelli et al., 2025), containing biomedical titles and abstracts related to the gut microbiota and its effects on mental health. The collection ships training and development partitions; gold-standard annotations for the test partition were not publicly released, so all results reported here are computed on the official development partition and are therefore not directly comparable to the challenge leaderboard, which was scored on the held-out test partition.

After pre-processing, the development partition yields 20,227 scored instances over 15 predicates. Our pre-processing generates candidate entity pairs with context windows and assigns *NONE* to those without an annotated relation, so *NONE* is a negative class introduced here rather than a relation defined by the corpus. Two predicates, *affect* and *produced by*, are retained too rarely after entity-pair generation to be evaluated reliably and are excluded; macro F1 is therefore averaged over 15 of the 17 annotated predicates. These 15 are *strike, used by, is linked to, located in, change effect, target, interact, influence, impact, part of*, *change expression, is a, compared to, administered*, and *change abundance*. The label distribution is highly skewed, with a few frequent relations and a long tail of rare but biologically meaningful ones, which is why we emphasize macro-averaged metrics.

To test whether findings obtained on GutBrainIE hold more generally, we evaluate on four further relation corpora, chosen so that they differ from GutBrainIE and from each other along the dimensions most likely to matter: annotation scope, class balance, and what the relation labels encode.

**ChemProt** (Krallinger et al., 2017) annotates chemical-protein interactions in PubMed abstracts at the sentence level. Its ten relation types (regulator, upregulator, downregulator, agonist, antagonist, substrate, cofactor, modulator, part-of, and a negative class) name pharmacological relationships.

**BioRED** (Luo et al., 2022) annotates relations between genes, diseases, chemicals, variants, species and cell lines across whole PubMed abstracts, so relations may hold between mentions in different sentences. Its eight relation types include *Association*, a residual category whose boundary against *Positive_Correlation* is set by the annotation guideline rather than by a distinction in the world.

**BC8** (Islamaj et al., 2024) is the BioCreative VIII release of BioRED and shares its schema. We report it separately because it is a distinct evaluation set, and together with BioRED it indicates how stable the results are across two samples drawn under one annotation scheme.

**DDI-2013** (Herrero-Zazo et al., 2013) annotates drug-drug interactions at the sentence level. Its four types (EFFECT, MECHANISM, ADVISE, INT) describe how a sentence discusses an interaction rather than a property of the drugs, and every candidate pair is drug-drug. DDI-2013 is the most balanced corpus in our set and, like ChemProt, is annotated within single sentences, which is what allows it to separate explanations that are confounded on the other corpora (Section 6.4).

Table 2 summarizes the evaluation sets. The label distributions are skewed on all corpora and severely so on BioRED and BC8, which is why we report macro- as well as micro-averaged scores throughout.

**Table 2:** Evaluation sets for the four additional corpora.

Corpus	Scope	Types	Instances	Largest class
ChemProt	sentence	10	5,744	30.3%
BioRED	abstract	8	1,163	54.6%
BC8	abstract	7	1,091	50.4%
DDI-2013	sentence	4	979	36.8%

For each corpus we evaluate over the full annotated relation inventory rather than the subset scored by its associated shared task. Label spaces are therefore comparable across corpora in the sense that each is the corpus's own complete inventory, but results are not directly comparable to shared-task leaderboards.

*Scope of each analysis*. The corpora are not used symmetrically. RQ1 to RQ3, comprising the comparison across models against the supervised baseline, the shot sweep, the per-class breakdown and the out-of-schema analysis, are conducted on GutBrainIE alone, since they require many configurations per corpus. RQ4 uses all five corpora and asks whether those findings generalize; the zero-shot and 10-shot results for every model on every corpus are reported there (Table 3).

**Table 3:** Zero-shot vs. 10-shot retrieval-augmented performance for all models on all five corpora.

Dataset	Model	Macro F1			Micro F1		
		0-shot	10-shot	Δ	0-shot	10-shot	Δ
GutBrainIE	DeBERTa+CNN (supervised)	—	0.440	—	—	0.562	—
	Llama-3.1-8B	0.004	0.211	+0.207	0.084	0.370	+0.286
	Gemma3:12B	0.028	0.318	+0.290	0.140	0.658	+0.518
	Qwen3:14B non-reas	0.105	0.767	+0.662	0.122	0.754	+0.632
	Qwen3:14B reasoning	0.031	0.732	+0.701	0.099	0.742	+0.643
ChemProt	DeBERTa+CNN (supervised)	—	0.565	—	—	0.630	—
	Llama-3.1-8B	0.243	0.439	+0.196	0.215	0.501	+0.286
	Gemma3:12B	0.386	0.487	+0.101	0.348	0.539	+0.191
	Qwen3:14B non-reas	0.450	0.579	+0.129	0.427	0.647	+0.220
	Qwen3:14B reasoning	0.449	0.565	+0.116	0.419	0.622	+0.203
BioRED	DeBERTa+CNN (supervised)	—	0.310	—	—	0.584	—
	Llama-3.1-8B	0.148	0.185	+0.037	0.281	0.411	+0.130
	Gemma3:12B	0.174	0.217	+0.043	0.397	0.428	+0.031
	Qwen3:14B non-reas	0.349	0.383	+0.034	0.496	0.419	**–0.077**
	Qwen3:14B reasoning	0.353	0.377	+0.024	0.504	0.445	**–0.059**
BC8	DeBERTa+CNN (supervised)	—	0.265	—	—	0.554	—
	Llama-3.1-8B	0.169	0.327	+0.158	0.269	0.340	+0.071
	Gemma3:12B	0.161	0.188	+0.027	0.346	0.406	+0.060
	Qwen3:14B non-reas	0.239	0.382	+0.143	0.456	0.427	**–0.029**
	Qwen3:14B reasoning	0.298	0.326	+0.028	0.483	0.449	**–0.034**
DDI-2013	DeBERTa+CNN (supervised)	—	0.842	—	—	0.886	—
	Llama-3.1-8B	0.225	0.454	+0.229	0.377	0.539	+0.162
	Gemma3:12B	0.451	0.609	+0.158	0.550	0.656	+0.106
	Qwen3:14B non-reas	0.351	0.546	+0.195	0.376	0.556	+0.180
	Qwen3:14B reasoning	0.343	0.561	+0.218	0.354	0.573	+0.219

Δ is the change from zero-shot to 10-shot. The supervised DeBERTa+CNN baseline is shown for reference. Retrieval yields large gains on GutBrainIE and ChemProt, none on BioRED and BC8, where it degrades the strongest model, and large gains on DDI-2013 that still leave every model far below the supervised baseline. Bold values indicate a decrease in performance (negative) from zero-shot to 10-shot.

### 4.2 Pre-processing pipeline

To prepare the data for relationship classification, annotated abstracts and titles are systematically processed to extract entity pairs, generate context windows, and map character offsets necessary for sentence-level cross-sections. Sentence segmentation is performed using spaCy. The workflow reads training and development data from JSON files, containing head and tail entities along with their annotated relations.

For supervised learning, representative negative samples (e.g., *NONE*) are included and downsampled to reduce class imbalance. After processing, cross-sentence entity pairs and their context windows are organized for both baseline and RAG-augmented LLM experiments.

### 4.3 Baseline model comparison

To evaluate the performance of retrieval-augmented LLM pipelines, we conducted a head-to-head comparison with a transformer-based neural model, DeBERTa+CNN. We adopt this as a representative strong supervised baseline rather than as an attempt at state of the art. DeBERTa-v3 is a competitive transformer encoder for biomedical text and a CNN classification head over entity-aware pooling is a standard, well-understood choice for relation typing, so the pairing is a familiar and reproducible reference point. The comparison of interest in this study is between two paradigms, supervised fine-tuning on a fixed annotated set and retrieval-augmented prompting over that same set, and that comparison is cleanest when the supervised side is a single, transparent, widely used model trained on identical data. While tuned ensembles of domain-pre-trained encoders, such as those submitted to the BioCreative VIII BioRED track (Section 2), achieve higher scores on this corpus, they obscure paradigm comparisons by introducing variables like ensembling, data augmentation, and task-specific engineering.

Both approaches draw on the same annotated pool, and differ in how they use it. The supervised baseline fits its parameters to the full training partition, whereas the retrieval-augmented pipelines select *k* neighbors per query by embedding similarity, without reference to their relation labels, and perform no parameter updates; the model conditions on those *k* demonstrations alone at inference. GutBrainIE is annotated at small scale, the training partition comprising a limited set of expert-annotated abstracts from which candidate entity pairs are generated mechanically, so the scarce resource is annotation effort rather than the number of derived training instances. Since expert annotation is the primary resource constraint in this domain, the practical challenge lies in determining how to deploy a limited pool of labeled data to maximize relation classification performance, a comparison that forms the core of this study.

Each document is tokenized and embedded using the DeBERTa-v3-base transformer, which generates context-aware token embeddings that capture semantic and syntactic information across the input text. To further capture local semantic patterns relevant to entity interactions, the embeddings are passed through a convolutional layer consisting of 256 filters with a kernel size of three. The resulting feature vectors are fed into a classification layer, producing an 18-dimensional output corresponding to the relation types defined in the GutBrainIE challenge. During evaluation, metrics are computed over the 15 relation labels that appear in the development partition; the three training-only labels (NONE, affect, produced by) have zero test instances and therefore do not contribute to test-time scores.

The network is trained using a weighted cross-entropy loss with balanced class weights to address prevalent class imbalance in the dataset. This setup provides a strong transformer-based benchmark against which the performance of the RAG pipelines and LLM-based models can be systematically compared.

For completeness we report the full training configuration. The encoder is DeBERTa-v3-base and is not frozen; token embeddings pass through a single 1D convolutional layer (768 → 256 channels, kernel size 3), followed by entity-aware mean pooling over the head and tail entity spans, concatenation of the pooled representations, and a linear classifier. The complete model has approximately 184M trainable parameters. Optimization uses AdamW with a learning rate of 5 × 10^−5^, a cosine learning-rate schedule, and a batch size of 16. Training stops early on validation micro-F1. We do not use k-fold cross-validation: each reported baseline score comes from a single training run with random seed 42, and the implications of reporting single runs are discussed in Section 6.5.

The same baseline architecture and training configuration are used on every corpus, with only the size of the output layer changing to match each relation inventory. Both the baseline and the RAG pipelines are evaluated on all five corpora described in Section 4.1. This comparison enables a clear evaluation of the strengths and limitations of both approaches for real-world biomedical relation classification tasks. The full implementation of the RAG4RE pipeline and experimental setup is available at the GitHub repository (Jannat, 2025).

### 4.4 Evaluation metrics

To assess the performance of both the RAG-augmented LLMs and the DeBERTa+CNN baseline for biomedical relation classification, we use a combination of standard metrics that capture performance across frequent and rare relation classes:

- Precision, recall, and F1-score: standard classification metrics, where F1 is the harmonic mean of precision and recall.
- Macro-averaged metrics: computed by averaging the metric across all relation classes. Macro F1 gives equal weight to each class, which is particularly important for biomedical datasets where minority classes may be highly significant.
- Micro-averaged metrics: computed globally by counting total true positives, false positives, and false negatives across all classes. Micro F1 emphasizes performance on the most frequent classes.
- Out-of-schema labels: for LLM-based models, we track predictions of labels that fall outside the defined relation set for the corpus. This measure helps quantify over-generation or incorrect inference, especially relevant in RAG few-shot settings.

These metrics provide complementary insights: macro metrics highlight the model's ability to handle rare but critical relations, while micro metrics reflect overall prediction quality and alignment with high-frequency labels.

### 4.5 Experimental protocol

To ensure reproducibility and a fair comparison between the baseline and RAG-augmented LLMs, the following experimental procedures were followed:

- **Dataset:** experiments were conducted on the BioASQ Conference and Labs of the Evaluation Forum (CLEF) 2025 GutBrainIE dataset (Martinelli et al., 2025), containing PubMed abstracts with annotated biomedical entities and relation labels.
- **Pre-processing:** all abstracts were pre-processed by tokenization, lowercasing, and entity annotation alignment. For RAG experiments, passages were chunked to ensure that each retrieval node contained at most 200 tokens while preserving entity relationships.
- **Retrieval-augmented generation (RAG) setup:**

– Document embeddings were created using the all-MiniLM-L6-v2 model and stored in Qdrant via LlamaIndex.
– For each test query, *n* most relevant nodes were retrieved using vector similarity.
– Retrieved nodes were formatted into structured few-shot prompts, containing passage text, head and tail entities, labels, and relation information, and fed into the LLMs.

- **Few-shot experiments:** to investigate the effect of in-context examples, experiments varied the number of retrieved examples (shots) from 0 to 60. Both reasoning and non-reasoning LLMs were evaluated across these shot counts.
- **Models evaluated:**

– DeBERTa+CNN baseline (supervised learning).
– Four RAG-augmented LLM variants:

* Llama-3.1-8B-Instruct (Non-Reasoning)
* Gemma3:12B (Reasoning)
* Qwen3:14B (Non-Reasoning)
* Qwen3:14B (Reasoning)

- **Inference:** each model produced predictions in a standardized JSON format containing the predicted relation and reasoning (if available). For the LLMs, out-of-schema labels (relations not present in the annotated dataset) were also tracked to quantify over-generation.
- **Generation parameters and software environment:** all large language models were served locally with Ollama (v0.30.10) and queried through LlamaIndex (v0.13.3) using an 8,000-token context window. Decoding used each model's default Ollama configuration; for the primary Qwen3:14B model this corresponds to the qwen3:14b build with Q4_K_M quantization and a sampling temperature of 0.8. We did not override the temperature, maximum output length, or random seed; the full configuration is specified here to support reproduction. Document embeddings were generated with all-MiniLM-L6-v2 (384-dimensional) via sentence-transformers (v5.1.0) and indexed in Qdrant (qdrant-client v1.15.1) for cosine-similarity retrieval. Evaluation metrics were computed with scikit-learn (v1.6.1) under Python 3.12.
- **Evaluation procedure:** model predictions were compared to ground truth using macro- and micro-precision, recall, and F1-score. Per-class F1 scores were also computed to assess performance on both frequent and rare relations. Experiments were repeated across multiple shots to analyze the effect of in-context examples on model performance and out-of-schema behavior.

## 5 Results

The performance of the retrieval-augmented LLM pipelines was evaluated against the DeBERTa+CNN baseline for relationship classification on the biomedical dataset. Table 4 summarizes the macro- and micro-averaged precision, recall, and F1 scores for all models.

**Table 4:** Comparison of DeBERTa+CNN and LLM models with 10 shots on macro and micro metrics.

Model	Macro P	Macro R	Macro F1	Micro P	Micro R	Micro F1
Deberta+CNN (baseline)	0.518	0.433	0.440	0.658	0.490	0.562
Llama-3.1-8B-Instruct	0.290	0.232	0.211	0.370	0.370	0.370
Gemma3:12B	0.354	0.323	0.318	0.658	0.658	0.658
Qwen3:14B non-reasoning	0.800	0.784	**0.767**	0.753	0.753	**0.753**
Qwen3:14B reasoning	0.723	0.742	**0.732**	0.742	0.742	**0.742**

Bold values indicate retrieval-augmented models that exceed the DeBERTa + CNN supervised baseline on both macro and micro F1.

Macro-averaged metrics are emphasized because they reflect the model's performance across all relation classes, including minority classes, which often carry critical biomedical meaning and are particularly affected by class imbalance. Micro-averaged metrics provide an overall performance measure across all instances.

As shown in Table 4, on GutBrainIE the retrieval-augmented Qwen3:14B configurations exceed the DeBERTa+CNN baseline on both macro and micro F1 at 10 shots, while the two smaller models do not.

## 6 Discussion

This section discusses the experimental results in the context of the research questions. The comparison between RAG-based LLMs and the DeBERTa + CNN baseline provides insights into the effectiveness of retrieval-augmented prompting, reasoning capabilities of different models, and the impact of varying the number of in-context examples. We structure the discussion around the four research questions:

- RQ1: how does the performance of RAG-based biomedical relation classification vary across LLMs relative to a DeBERTa + CNN baseline in terms of precision, recall, and F1-score?
- RQ2: what is the impact of increasing in-context examples on performance, and where do diminishing returns occur for different LLMs?
- RQ3: to what extent does retrieval augmentation reduce out-of-schema or incorrect relation labels in LLM-based classification?
- RQ4: does the benefit of retrieval augmentation generalize across corpora with different relation schemas, and what distinguishes corpora where it helps from those where it does not?

Each subsection below addresses one research question, linking the quantitative results presented in Section 5 to the broader implications for biomedical relationship classification and model design.

### 6.1 Performance comparison across models (RQ1)

Table 4 summarizes macro- and micro-averaged precision, recall, and F1 scores for all models evaluated on the GutBrainIE biomedical dataset. The DeBERTa+CNN baseline achieved a macro F1 of 0.440 and micro F1 of 0.562, serving as a reference point for assessing the benefits of retrieval-augmented LLMs.

Among the LLMs with retrieval augmentation:

- Qwen3:14B (Non-Reasoning) achieved the highest macro F1 score of 0.767 and micro F1 of 0.753.
- Qwen3:14B (Reasoning) closely followed, with a macro F1 of 0.732 and micro F1 of 0.742.
- Gemma3:12B reached a macro F1 of 0.318 and micro F1 of 0.658, showing moderate improvement in some metrics relative to the baseline.
- Llama-3.1-8B-Instruct underperformed relative to the baseline, with a macro F1 of 0.211 and micro F1 of 0.370.

Table 5 presents per-class predicted frequencies for each model compared to ground-truth annotations, highlighting differences in how models distribute predictions across relation types. Qwen models closely match the frequency of high-occurrence classes such as *is a, is linked to*, and *located in*. In contrast, Llama-3.1-8B-Instruct often over-predicts low-frequency classes and under-predicts medium-frequency ones, while Gemma3:12B shows moderate alignment but under-predicts several low-frequency relations. This miscalibration explains the macro-averaged gap: because macro-F1 weights every relation equally, a model that floods a handful of labels, Llama predicts *is linked to* over five times as often as it occurs, collapses on the long tail of rarer relations and is penalized far more in macro-F1 than in micro-F1. The Qwen models track the ground-truth distribution closely, which is reflected in their substantially higher macro scores.

**Table 5:** Per-class predicted frequencies for all models compared to ground truth.

Class label	Ground truth	Gemma3:12B	Llama-3.1-8B-Instruct	Qwen3:14B non-reasoning	Qwen3:14B reasoning
is linked to	1,126	3,348	5,754	2,568	2,816
compared to	500	479	64	626	670
located in	1,865	1,930	1,845	2,207	2,302
change abundance	1,568	281	303	746	628
is a	4,788	3,690	1,229	3,648	3,682
target	1,273	958	1,386	1,091	1,052
strike	612	2,171	37	356	365
interact	2,165	1,521	255	1,123	1,267
impact	927	1,469	678	1,608	1,052
influence	1,955	2,771	4,313	3,124	3,005
administered	974	1,352	1,342	777	766
change expression	494	334	414	391	376
used by	225	240	227	117	157
part of	1,214	1,169	1,295	1,145	1,164
change effect	541	425	748	585	568

Overall, on GutBrainIE the Qwen3:14B models outperform the DeBERTa+CNN baseline on both macro and micro metrics, with the macro margin indicating better coverage of rare relations. Whether this ordering holds on other corpora is taken up in RQ4. Smaller models or models with weaker retrieval augmentation exhibit inconsistent alignment with ground truth, underscoring the importance of retrieval augmentation and model scale in biomedical relation classification.

### 6.2 Impact of in-context examples (shots) on model performance (RQ2)

To evaluate the impact of the number of retrieved examples on LLM performance, we conducted ablation experiments with Qwen3:14B reasoning and non-reasoning variants across 0–60 in-context examples (“shots”). Table 6 and Figure 2 summarize macro F1 scores and the number of out-of-schema labels at each shot count.

**Table 6:** Macro F1 and out-of-schema label counts for Qwen3:14B on GutBrainIE across in-context example counts.

#Shots	Non-reasoning macro F1	#Out-of-schema	Reasoning macro F1
0	0.105	39	0.031
5	0.607	9	—
10	0.767	5	0.732
15	0.799	2	—
20	0.852	1	—
25	0.877	1	0.815
30	0.885	1	—
**35**	**0.892**	**0**	—
40	0.896	4	—
45	0.905	3	—
50	0.899	2	—
55	0.903	10	—
60	0.902	16	—

The finer-grained sweep was run for the non-reasoning variant only; the reasoning variant was evaluated at 0, 10 and 25 shots, each averaged over three runs (Section 6.5). Out-of-schema counts were not recorded for the reasoning runs. Bold values indicate the shot count (35) at which out-of-schema prediction first reach zero.

**Figure 2:**
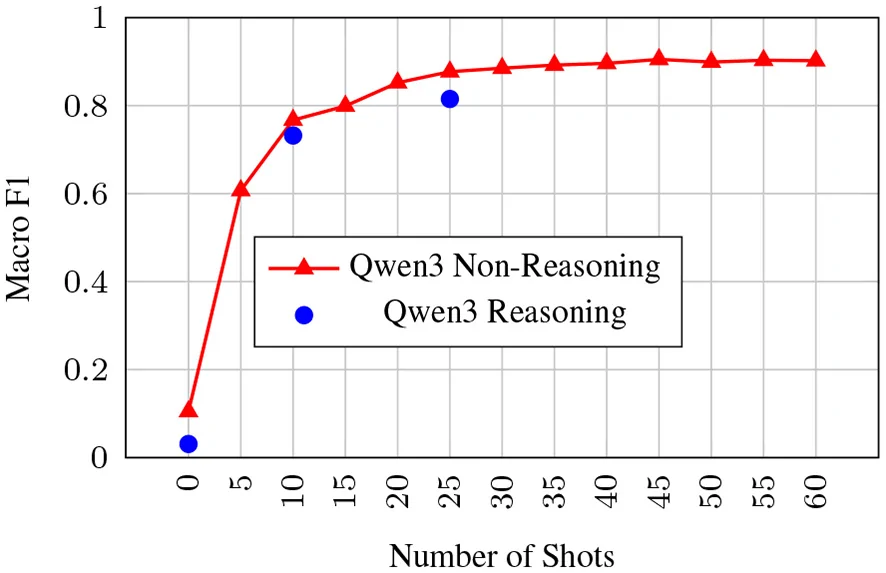
Macro F1 scores for Qwen3:14B reasoning and non-reasoning variants across different numbers of in-context examples (shots).

The non-reasoning variant shows rapid improvement in macro F1 during the first 10–25 shots, and beyond 35–40 shots macro F1 begins to plateau, indicating diminishing returns from additional in-context examples. The reasoning variant, evaluated at 0, 10 and 25 shots, follows the same trajectory over that range. Out-of-schema labels decrease sharply with increasing shots, reaching near-zero at 25–35 shots, and increase slightly at higher shot counts (Figure 3). This reduction demonstrates that in-context examples provide grounding that improves prediction reliability, particularly for low-frequency or ambiguous relations.

**Figure 3:**
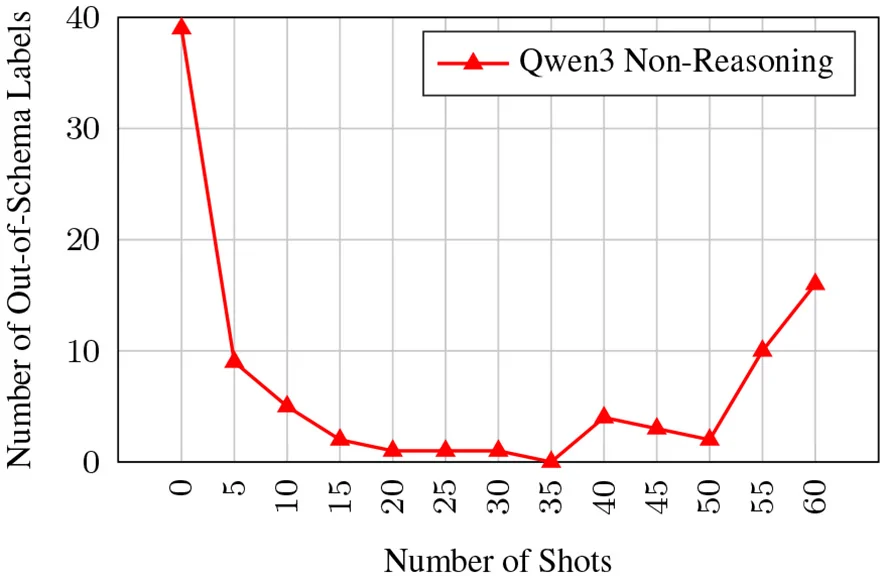
Number of out-of-schema labels for Qwen3:14B non-reasoning on GutBrainIE across shot counts. Out-of-schema counts were not recorded for the reasoning variant.

In summary, increasing the number of in-context examples significantly boosts performance up to 35–40 shots, after which gains plateau. Retrieval-augmented prompts further reduce out-of-schema predictions, especially for low-frequency or semantically ambiguous classes, highlighting the importance of both context and grounding for robust biomedical relation classification.

A per-class breakdown (Table 7) makes the same trend visible at the relation level: retrieval augmentation lifts F1 across nearly all relation types, with the steepest gains within the first 10 shots and a plateau by roughly 35-40. The zero-shot column (0) confirms that, without retrieved examples, the model performs poorly across every class, isolating the contribution of retrieval.

**Table 7:** Per-class F1 for Qwen3:14B *non-reasoning* on GutBrainIE across in-context example counts (shots); the 0-shot column is the zero-shot setting.

Relation	0	5	10	15	20	25	30	35	40	45	55	60
administered	0.112	0.779	0.883	0.915	0.933	0.947	0.951	0.959	0.966	**0.967**	0.960	0.947
change abundance	0.068	0.239	0.443	0.489	0.534	0.576	0.572	**0.616**	0.535	0.573	0.566	0.603
change effect	0.043	0.663	0.837	0.903	0.941	0.976	0.967	0.974	0.983	**0.985**	0.982	0.976
change expression	0.014	0.662	0.823	0.887	0.925	0.933	0.933	0.967	0.957	0.968	**0.979**	0.961
compared to	0.025	0.618	0.787	0.824	0.896	0.921	0.944	0.964	0.970	**0.981**	0.980	0.980
impact	0.096	0.452	0.630	0.797	0.848	0.888	0.888	0.910	**0.912**	0.899	0.908	0.892
influence	0.050	0.595	0.691	0.722	0.749	0.766	0.766	0.753	0.778	**0.783**	0.759	0.768
interact	0.016	0.461	0.603	0.697	0.691	0.732	0.730	0.710	0.750	**0.751**	0.709	0.713
is a	0.166	0.694	0.820	0.889	0.930	0.943	0.959	0.962	0.976	0.977	0.981	**0.982**
is linked to	0.109	0.419	0.564	0.617	0.648	0.661	0.701	0.718	0.703	0.714	0.715	**0.722**
located in	0.174	0.823	0.891	0.910	0.927	0.942	0.947	**0.951**	0.957	0.963	0.967	0.963
part of	0.059	0.802	0.905	0.935	0.968	0.978	0.979	0.985	0.987	0.992	**0.989**	0.987
strike	0.000	0.436	0.717	0.818	0.832	0.846	0.860	0.858	0.878	0.901	0.914	**0.919**
target	0.088	0.628	0.807	0.875	0.933	0.944	0.951	0.958	0.974	0.971	**0.975**	0.963
used by	0.243	0.846	0.978	0.971	0.991	**0.996**	0.991	0.993	0.989	0.984	0.984	0.986

Bold indicates the highest F1 score achieved for each relation across the shot sweep.

### 6.3 Effect of retrieval augmentation on out-of-schema or incorrect labels (RQ3)

To assess the impact of retrieval augmentation, we analyzed out-of-schema and incorrect relation predictions for Qwen3:14B non-reasoning across the full shot sweep; out-of-schema counts were not recorded for the reasoning variant. Table 6 and Figure 3 illustrate the evolution of out-of-schema predictions as the number of retrieved examples increases. Retrieval augmentation substantially reduces them: the model emits 39 out-of-schema labels at zero shots, and with 20–35 retrieved examples this drops to zero or one.

The effect does not require explicit reasoning: the non-reasoning variant reaches zero out-of-schema predictions at 35 shots, which is consistent with retrieval supplying the label inventory and output format directly rather than through any reasoning step. Diminishing gains are observed at higher shot counts; beyond 35–40 shots, out-of-schema predictions increase slightly, likely due to over-extension of retrieved context, though the overall numbers remain far lower than zero-shot or non-RAG conditions. Low-frequency and semantically ambiguous classes (e.g., “administered to,” “strike”) show the most notable decreases, indicating that retrieval provides disambiguating context.

In summary, retrieval augmentation markedly reduces out-of-schema and incorrect relation predictions for the non-reasoning variant across the full shot sweep. Near-zero out-of-schema predictions are achieved with 25–35 retrieved examples, particularly benefiting low-frequency and ambiguous classes, demonstrating that retrieval provides essential grounding for accurate biomedical relation classification.

### 6.4 Generalization across relation schemas (RQ4)

Extending the evaluation beyond GutBrainIE reveals that the benefit of retrieval augmentation is not uniform across corpora. Table 8 shows two distinct outcomes. On GutBrainIE and ChemProt the best retrieval-augmented configuration exceeds the supervised baseline on both macro and micro F1. On BioRED and its BioCreative VIII release (BC8) the retrieval-augmented models improve macro F1 but fall below the baseline on micro F1, and on BioRED retrieval reduces micro F1 relative to the zero-shot setting (Table 3).

**Table 8:** Best retrieval-augmented configuration against the supervised baseline on all five corpora.

Dataset	Model	Macro P	Macro R	Macro F1	Micro P	Micro R	Micro F1
GutBrainIE	DeBERTa+CNN (supervised)	0.518	0.433	0.440	0.658	0.490	0.562
	Qwen3:14B non-reasoning (25-shot)	0.904	0.884	**0.877**	0.866	0.866	**0.866**
	Qwen3:14B reasoning (25-shot)	0.840	0.822	0.815	0.856	0.856	0.856
ChemProt	DeBERTa+CNN (supervised)	0.552	0.659	0.565	0.630	0.630	0.630
	Qwen3:14B non-reasoning (25-shot)	0.570	0.708	0.591	0.658	0.658	0.658
	Qwen3:14B reasoning (25-shot)	0.744	0.730	**0.735**	0.795	0.795	**0.795**
BioRED	DeBERTa+CNN (supervised)	0.311	0.329	0.310	0.584	0.584	**0.584**
	Qwen3:14B non-reasoning (25-shot)	0.435	0.523	0.407	0.476	0.476	0.476
	Qwen3:14B reasoning (25-shot)	0.466	0.549	**0.438**	0.457	0.457	0.457
BC8	DeBERTa+CNN (supervised)	0.280	0.253	0.265	0.554	0.554	**0.554**
	Qwen3:14B non-reasoning (25-shot)	0.365	0.387	**0.334**	0.501	0.501	0.501
	Qwen3:14B reasoning (25-shot)	0.348	0.375	0.321	0.499	0.499	0.499
DDI-2013	DeBERTa+CNN (supervised)	0.879	0.821	**0.842**	0.886	0.886	**0.886**
	Qwen3:14B non-reasoning (25-shot)	0.667	0.485	0.548	0.576	0.576	0.576
	Qwen3:14B reasoning (25-shot)	0.622	0.496	0.543	0.597	0.597	0.597

Best macro and micro F1 per corpus are in bold. Llama-3.1-8B and Gemma3:12B were not run at 25 shots and are reported at 10 shots in Table 3.

The divergence between macro and micro F1 is measurable for this split. BioRED and BC8 are dominated by a single relation type, *Association*, which accounts for over half of the instances. A supervised model learns a baseline pattern from thousands of training examples. The micro F1 metric rewards this model because it gives equal importance to every single data point. The retrieval-augmented models distribute predictions more evenly across the label set, which raises macro F1 by improving recall on rare relations while lowering overall accuracy.

One explanation consistent with this pattern concerns what the relation labels encode. The GutBrainIE predicates (*located in, influence, part of* ) and the ChemProt classes (regulator, upregulator, downregulator, agonist, antagonist, substrate, cofactor, modulator) describe relationships that are easily inferred and distinguishable from each other in general and biomedical world knowledge. In contrast, the boundary between *Association* and *Positive_Correlation* in BioRED is an annotation convention rather than a distinction about the world. A small number of in-context demonstrations appears sufficient to convey a label inventory and an output format, but not to induce a guideline-specific boundary, which supervised training over thousands of annotated examples can learn.

Across GutBrainIE, ChemProt, BioRED and BC8 this explanation is confounded: label semantics co-vary with class imbalance and with whether relations are annotated within or across sentence boundaries, and any of the three could account for the split. DDI-2013 separates them. Its four categories (EFFECT, MECHANISM, ADVISE, INT) describe how a sentence discusses an interaction rather than a property of the drugs themselves, so its labels are annotation conventions; but it is the most balanced corpus in our set (36.8%, 30.8%, 22.6% and 9.8% of the test instances), and it is annotated within single sentences, as ChemProt is. It therefore holds imbalance and annotation scope fixed while varying what the labels encode.

The outcome follows the label-semantics account. The supervised baseline reaches 0.842 macro and 0.886 micro F1 on DDI-2013, while the best retrieval-augmented configuration reaches 0.561 macro and 0.597 micro (Table 8). This is the only corpus of the five on which the baseline is stronger on both measures, so the macro/micro divergence that accounts for BioRED and BC8 cannot account for it. It is also the reverse of ChemProt, which is matched to DDI-2013 in annotation scope and comparable in balance, and on which the retrieval-augmented model wins by 0.170 macro F1. The distinguishing property that remains is that the ChemProt classes (regulator, upregulator, downregulator, agonist, antagonist, substrate, cofactor, modulator) name pharmacological relationships an LLM encounters during pre-training, whereas the DDI-2013 categories are defined by the annotation guideline.

The model's poor performance on DDI-2013 is not caused by a failure to find or retrieve the data. Retrieval improves every model substantially on this corpus, by 0.16 to 0.23 macro F1 from the zero-shot to the 10-shot setting, which is larger than the corresponding gains on ChemProt (Table 3). What retrieval does not do is close the gap to a model trained on the guideline. DDI-2013 also removes the entity-type shortcut discussed below, since every candidate pair is drug-drug and a majority-predicate-per-type-pair baseline therefore reduces to the majority class at 36.8%. The corpus is not intrinsically hard: the supervised model solves it well. It is hard for in-context learning specifically, which is the distinction the earlier corpora could not isolate.

### 6.5 Limitations

Several limitations constrain the interpretation of these results.

#### 6.5.1 Evaluation partition

The gold-standard annotations for the GutBrainIE 2025 test partition were not publicly released. All GutBrainIE results are therefore computed on the official development partition and are not directly comparable to the challenge leaderboard.

#### 6.5.2 Run-to-run variance

Decoding used each model's default sampling configuration. The 0-, 10- and 25-shot conditions were repeated three times, giving a standard deviation of 0.020-0.031 in macro F1. The remaining shot counts are single runs, and differences of this magnitude or smaller should not be interpreted as meaningful. In particular, the differences between shot counts above 25 fall well within this range, so while performance clearly saturates, we do not claim to locate the saturation point precisely.

#### 6.5.3 Schema constraints on GutBrainIE

The GutBrainIE relation schema constrains which predicates are admissible for each pair of entity types. Because our task formulation supplies gold entity pairs together with their types, a baseline that predicts the majority predicate for each entity-type pair, without reading the text at all, is strongly favored by the schema: of the 45 distinct entity-type pairs in the annotated data, 42 admit exactly one predicate. This property is specific to the GutBrainIE schema, which constrains the predicates admissible for each head-tail type pair; the same baseline is uninformative on DDI-2013, where every candidate pair is drug-drug and it reduces to the majority class. The same caution applies to ChemProt, where macro F1 averages over ten relation types, two of which (cofactor and modulator) have 25 instances each against 1,743 for the most frequent type.

#### 6.5.4 Pre-processing coverage

Entity-pair generation with context windows does not retain all annotated relations. Two predicates, *affect* and *produced by*, are reduced to too few instances to evaluate reliably and are excluded from the reported label set. Macro F1 for GutBrainIE is therefore averaged over 15 of the 17 annotated predicates.

#### 6.5.5 Retrieval and train/test overlap

Because demonstrations are retrieved from the training pool by semantic similarity, a test instance with a near-duplicate in that pool could be supplied with its own answer rather than with an analogous example. We therefore checked whether the retrieval pool and the evaluation set share source documents. They do not: the sets of source article identifiers are disjoint on every corpus, so no retrieved demonstration originates from a document that also contributes an evaluation instance. This rules out the most common route to this problem, in which a corpus is partitioned at the instance level from a shared pool of abstracts and sibling instances from one document land on both sides.

It does not establish that no evaluation instance has a paraphrase somewhere in the retrieval pool, and we do not claim that. We note two observations that bear on how much such paraphrases could be contributing. First, retrieval helps least on the two corpora where sentences are most formulaic (BioRED and BC8), and on those corpora it reduces micro F1 relative to the zero-shot setting, which is the opposite of what answer-copying would produce. Second, on DDI-2013 retrieval produces large gains and still leaves every model far below the supervised baseline, so the demonstrations are not carrying labels across.

*Scope of the reasoning comparison*. The Qwen3:14B reasoning variant was evaluated at 0, 10 and 25 shots, each averaged over three runs. The finer-grained shot sweep from 0 to 60 shots is reported for the non-reasoning variant only, and out-of-schema counts were recorded for that variant only. Comparisons between the two variants are therefore restricted to the three shot counts at which both were run.

## 7 Conclusion

This work evaluates retrieval-augmented large language models for biomedical relation classification. On GutBrainIE, retrieval-augmented prompting of Qwen3:14B exceeds a supervised DeBERTa+CNN baseline on both macro and micro F1; the gains concentrate in the first ten retrieved examples and saturate by roughly 25; and predictions falling outside the defined relation set drop to at most one by 20 to 35 shots. The smaller general-purpose models do not reach the baseline on macro F1.

Repeating the comparison on four further corpora shows that these findings do not generalize uniformly. On ChemProt the retrieval-augmented configuration again exceeds the baseline. On BioRED and its BioCreative VIII release it improves macro F1, reflecting better coverage of rare relations, but falls below the baseline on micro F1, where a supervised model trained on thousands of examples exploits a dominant majority class more effectively. On DDI-2013 the baseline is stronger on both measures by a wide margin. Because DDI-2013 is the most balanced corpus in the set and is annotated within single sentences, as ChemProt is, its class distribution and annotation scope cannot explain the reversal; what separates it from ChemProt is that its categories are defined by an annotation guideline rather than by pharmacology. Retrieval still improves every model on DDI-2013, so the limitation is not in retrieval but in what a small number of demonstrations can convey.

Model capability matters, but not uniformly. The smaller general-purpose models (Llama-3.1-8B, Gemma3:12B) underperform the baseline throughout. Between the two Qwen3:14B variants the comparison is inconsistent across corpora: the non-reasoning variant is stronger on GutBrainIE while the reasoning variant is stronger on ChemProt, and with single runs at most configurations we do not draw a general conclusion about explicit reasoning. The reasoning variant does produce rationales alongside its predictions, which makes individual decisions easier to inspect.

Varying the number of retrieved examples shows that performance improves quickly within the first 10 in-context examples and continues to improve to 25. Beyond that point differences fall within the run-to-run standard deviation observed across replicated measurements, so we report saturation without claiming a precise saturation point. Retrieval augmentation also reduces predictions falling outside the defined relation set, most sharply at low shot counts.

Overall, retrieval-augmented prompting is a strong choice for biomedical relation classification when the label set is well covered by the retrieved demonstrations, and a supervised model remains preferable when a single relation type dominates the distribution. Characterizing which property of a corpus determines this is, we suggest, a more useful question for practitioners than whether retrieval augmentation helps in general.

## 8 Future work

Future research will explore extending retrieval-augmented LLMs for biomedical relation classification in several directions:

- **Ablation studies on Qwen3:14B models:** further experiments will systematically analyze the non-reasoning variant, which achieved high macro and micro F1 scores, to better understand the impact of reasoning components on RAG-based classification performance.
- **Iterative prompt tuning and temperature control:** exploring prompt optimization and adjusting generation parameters for both reasoning and non-reasoning models can help maximize performance, especially for challenging or rare biomedical relations.
- **Extension to named entity recognition and GraphRAG:** incorporating entity-centric document retrieval and graph-based representations can improve classification of complex biomedical relationships, allowing models to handle richer context and more intricate interaction patterns.
- **Learning annotation conventions without full supervision:** the corpora on which retrieval augmentation falls short are those whose label boundaries are defined by an annotation guideline rather than by biomedical knowledge. Supplying the guideline text itself alongside retrieved demonstrations, or combining a small amount of supervised adaptation with retrieval, would test whether that gap can be closed without full fine-tuning.
- **Domain-specific and newer LLMs:** a systematic comparison against recent biomedical-specialized LLMs, controlling for model scale and generation, would clarify whether domain-adapted pre-training helps once general capability is matched.
- **End-to-end extraction:** extending the pipeline from relation classification given gold entity pairs to joint entity-and-relation extraction would broaden its applicability to fully unstructured input.

## Data Availability

Publicly available datasets were analyzed in this study. This data can be found here: https://hereditary.dei.unipd.it/challenges/gutbrainie/2025/.
